# Diabetes mellitus: complicações associadas ao tempo de diagnóstico, plano de saúde, uso de serviços de saúde e uso de medicamentos, *Pesquisa Nacional de Saúde* 2019

**DOI:** 10.1590/0102-311XPT106624

**Published:** 2025-06-27

**Authors:** Veronica Batista Gomes Leitão, Lorena Goulart de Andrade, Aldiane Gomes de Macedo Bacurau, Daniela de Assumpção, Priscila Maria Stolses Bergamo Francisco

**Affiliations:** 1 Universidade Estadual de Campinas, Campinas, Brasil.; 2 Prefeitura Municipal de Campinas, Campinas, Brasil.

**Keywords:** Diabetes Mellitus, Complicações do Diabetes, Doenças Não Transmissíveis, Uso de Medicamentos, Estudos Transversais, Diabetes Mellitus, Diabetes Complications, Noncommunicable Diseases, Drug Utilization, Cross-Sectional Studies, Diabetes Mellitus, Complicaciones de la Diabetes, Enfermedades No Transmisibles, Utilización de Medicamentos, Estudios Transversales

## Abstract

O objetivo deste estudo é estimar a ocorrência de complicações do diabetes mellitus em adultos e sua associação com o tempo de diagnóstico da doença, posse de plano de saúde e uso de serviços de saúde e medicamentos. Trata-se de uma análise transversal de base populacional com dados da *Pesquisa Nacional de Saúde* (PNS) 2019. Foram considerados indivíduos com idade ≥ 40 anos (n = 54.987), estimada a prevalência de diabetes e suas complicações, e o uso de medicamentos. Verificaram-se as associações entre a ocorrência das complicações selecionadas e o tempo de diagnóstico do diabetes, posse de plano de saúde e utilização de serviços por meio de razão de prevalência bruta e ajustada, utilizando regressão de Poisson com variância robusta. A prevalência de diabetes nesse recorte foi de 13,4% (IC95%: 12,9-13,9), e de complicações foi de 31% (IC95%: 29,2-32,9). As mais prevalentes foram problemas de visão (29,6%; IC95%: 27,6-31,6) e renais (9,2%; IC95%: 8,2-10,4). As prevalências de todas as complicações aumentaram em relação ao tempo de diagnóstico da doença (p < 0,05). A prevalência de problemas de visão foi maior nos indivíduos sem um plano de saúde (32,7% *vs.* 21,5%; p < 0,001), e entre as pessoas que foram ao médico nos últimos seis meses (31,1% *vs.* 25,2%; p = 0,007) em relação às que foram de seis meses a menos de três anos. O uso de medicamentos entre aqueles com complicações foi de 93,3% (IC95%: 91,5-94,8), contudo mais de 1,5 milhão de pessoas neste grupo não faziam uso dos medicamentos prescritos. Faz-se necessário o fortalecimento das ações do Sistema Único de Saúde (SUS) - em especial exames de rastreamento para a identificação precoce do diabetes mellitus e suas complicações.

## Abstract (en)

This study aimed to estimate the occurrence of diabetes mellitus complications in adults and their association with the time since diagnosis, health insurance coverage, use of health services, and medication use. This is a population-based cross-sectional study using data from the 2019 *Brazilian National Health Survey* (PNS). Individuals aged ≥ 40 years (n = 54,987) were considered, and the prevalence of diabetes and its complications, as well as medication use, was estimated. Associations between the occurrence of the selected complications and the time since diabetes diagnosis, health insurance coverage, and health service use were examined using both crude and adjusted prevalence ratios, employing Poisson regression with robust variance. The prevalence of diabetes in the study population was 13.4% (95%CI: 12.9-13.9), and that of complications was 31% (95%CI: 29.2-32.9). The most prevalent complications were vision problems (29.6%; 95%CI: 27.6-31.6) and kidney problems (9.2%; 95%CI: 8.2-10.4). The prevalence of all complications increased with the time since diagnosis (p < 0.05). The prevalence of vision problems was higher among individuals without health insurance (32.7% vs. 21.5%; p < 0.001) and among those who had seen a doctor in the last six months (31.1% vs. 25.2%; p = 0.007) compared to those whose last visit was between six months and less than three years. Medication use among those with complications was 93.3% (95%CI: 91.5-94.8); however, more than 1.5 million people in this group were not using their prescribed medications. Strengthening actions by the Brazilian Unified National Health System (SUS) are necessary, particularly screening exams for the early identification of diabetes mellitus and its complications.

## Resumen

El objetivo de este estudio fue estimar la ocurrencia de complicaciones de la diabetes mellitus en adultos y su asociación con el tiempo de diagnóstico de la enfermedad, la posesión de seguro de salud, el uso de servicios de salud y de medicamentos. Este es un estudio transversal de base poblacional de la *Encuesta Nacional de Salud* (PNS) 2019. Participaron individuos ≥ 40 años (n = 54.987), y se estimó la prevalencia de diabetes y sus complicaciones, y el uso de medicamentos. Las asociaciones entre la ocurrencia de las complicaciones seleccionadas y el tiempo del diagnóstico de diabetes, la posesión de seguro de salud y el uso de servicios se comprobaron mediante una relación de prevalencia cruda y ajustada, con el uso de la regresión de Poisson con varianza robusta. La prevalencia de diabetes en la población de estudio fue del 13,4% (IC95%: 12,9-13,9); y las complicaciones del 31% (IC95%: 29,2-32,9). Las complicaciones más prevalentes fueron problemas de visión (29,6%; IC95%: 27,6-31,6) y problemas renales (9,2%; IC95%: 8,2-10,4). La prevalencia de todas las complicaciones aumentó en relación con el tiempo de diagnóstico de la enfermedad (p < 0,05). La prevalencia de problemas de visión fue mayor en individuos sin seguro de salud (32,7% vs. 21,5%; p < 0,001), y entre las personas que acudieron al médico en los últimos seis meses (31,1% vs. 25,2%; p = 0,007) en relación a las que pasaron de seis meses a menos de 3 años. Entre las personas con complicaciones, el uso de medicamentos fue del 93,3% (IC95%: 91,5-94,8), pero más de 1.5 millones de personas en este grupo no usaron los medicamentos que les había recetado. Es necesario promover las acciones del Sistema Único de Salud (SUS), especialmente las pruebas de cribado para la identificación precoz de la diabetes mellitus y sus complicaciones.

## Introdução

O diabetes mellitus é uma doença metabólica crônica, caracterizada por níveis elevados de glicose (açúcar) no sangue e que apresenta etiologia complexa e multifatorial, abrangendo desde fatores genéticos até ambientais ^1^ ^,^ ^2^. O tipo mais comum é o diabetes tipo 2, que ocorre devido a perda progressiva da secreção de insulina ou quando o organismo se torna resistente a esse hormônio ^1^ ^,^ ^2^ ^,^ ^3^. O diabetes mellitus pode progredir com complicações microvasculares (nefropatia, retinopatia, neuropatia ou amputações de membros inferiores) e macrovasculares (doença coronariana, acidente vascular cerebral ou doença vascular periférica), gerando impactos nos vasos sanguíneos, olhos e órgãos-alvo, como o coração, os rins e o cérebro ^1^ ^,^ ^4^. 

As complicações do diabetes mellitus contribuem para uma significativa morbimortalidade, resultando em elevados custos - tanto para as pessoas quanto para os sistemas de saúde - devido a fatores como amputações, aposentadorias antecipadas, perda de capacidade de trabalho em idades produtivas, absenteísmo laboral e despesas médico-hospitalares. Ainda, há aumento nos riscos de morte prematura (30-69 anos), tornando o diabetes mellitus uma das principais causas de óbitos em adultos no mundo ^5^ ^,^ ^6^ ^,^ ^7^. 

No Brasil, em 2013, a prevalência do diabetes mellitus era de 6,2%, e as complicações mais frequentes eram: os “problemas de visão” (31,9%), os circulatórios periféricos (13,7%), os nos rins (12,3%), os infartos/acidentes vasculares cerebrais (AVC, 7,6%) e as úlceras/feridas nos pés (6,1%) ^8^. Em 2019, a prevalência de diabetes na população adulta era de 7,7% e aumentou consideravelmente com a idade (especialmente após os 40 anos), atingindo 20,2% dos idosos ^1^. Esses percentuais podem ser ainda mais elevados, considerando a alta proporção de subnotificação de diabetes no país (42,5%), segundo estudo com dados da *Pesquisa Nacional de Saúde* (PNS) de 2013 ^8^. 

Mediante o diagnóstico da doença, para além das recomendações sobre mudanças no estilo de vida das pessoas com diabetes mellitus (prática de exercícios físicos, adequação da dieta etc.), deve-se considerar o uso de medicamentos como uma das principais estratégias terapêuticas de cuidado, e garantir o acesso a eles é essencial para o tratamento ^1^ ^,^ ^8^ ^,^ ^9^. 

Com o objetivo de ampliar o acesso a medicamentos essenciais, em 2004 o Ministério da Saúde criou o Programa Farmácia Popular do Brasil (PFPB), que passou oferecer à população mais uma alternativa de acesso aos remédios para tratamento de hipertensão e diabetes, cumprindo assim uma das principais diretrizes da Política Nacional de Assistência Farmacêutica (PNAF). Embora os valores gastos pelo Ministério da Saúde com o programa sejam muito superiores aos repassados aos municípios para a manutenção do Componente Básico da Assistência Farmacêutica (CBAF) - destinado à aquisição de todos os medicamentos no âmbito da atenção primária à saúde (APS), incluindo diabetes mellitus -, o programa Farmácia Popular se mostrou efetivo em reduzir internações hospitalares e mortalidade por doenças crônicas não transmissíveis (DCNT), tendo se firmado como importante avanço nas políticas de saúde do Brasil ^10^ ^,^ ^11^.

Portanto, a prevalência da doença e a importância de suas complicações para a qualidade de vida dos indivíduos acometidos e da família indicam a necessidade de investimentos e ações de prevenção, diagnóstico precoce, controle e cuidados longitudinais nos diferentes níveis de atenção à saúde, mas sobretudo na APS ^1^ ^,^ ^8^. O diabetes mellitus pode permanecer assintomático por longo tempo, e a investigação diagnóstica é frequentemente iniciada não pelos sintomas, mas pela presença de fatores de risco (como idade, hábitos alimentares não saudáveis, sedentarismo, obesidade, entre outros) ^3^ ^,^ ^12^. Algumas ações são importantes na prevenção do diabetes e de suas complicações, como o rastreamento de pessoas que possuem alto risco para desenvolver a doença (prevenção primária), para assim se iniciar os cuidados preventivos, e o rastreamento de quem tem diabetes e não sabe (prevenção secundária), a fim de se oferecer o tratamento em tempo oportuno. Nos casos já diagnosticados, é importante o monitoramento e o controle da glicemia, além de ações voltadas à educação em saúde para a prevenção de complicações e a manutenção da qualidade de vida das pessoas acometidas (prevenção terciária) ^12^.

Dados da PNS 2013 e do Programa de Melhoria do Acesso e da Qualidade na Atenção Básica (PMAQ-AB) de 2012 indicaram problemas na qualidade da atenção, na realização de exames e no acesso aos serviços de saúde na APS, que podem levar a desfechos adversos resultando na busca por emergências (27%) e internações hospitalares (15%) ^8^. 

Também o não comparecimento às consultas e a interrupção do tratamento medicamentoso - levando ao descontrole glicêmico - são fatores associados à internação hospitalar ou à busca por atendimentos de urgência e emergência, além de estarem relacionados ao maior desenvolvimento de complicações incapacitantes, como úlcera nos membros inferiores, pés diabéticos, amputações, retinopatia diabética, cegueira e insuficiência renal crônica, levando a internações prolongadas e recorrentes ^13^. Em um estudo realizado com cerca de 140 mil usuários de 87 unidades básicas de saúde (UBS) do Município do Rio de Janeiro, cerca de 22% dos pacientes informaram que costumavam deixar de tomar os medicamentos, outros 23,2% relataram que sobravam medicamentos; 20,7% haviam buscado serviços de emergência e 6% foram internados por complicações da hipertensão e do diabetes no ano anterior, com maior proporção de internação entre os indivíduos com diabetes ^13^. 

Essa associação entre ter o diagnóstico de diabetes mellitus há 10 anos ou mais e a piora na qualidade de vida foi reportada em estudo realizado em Minas Gerais, Brasil ^14^. Embora sejam encontrados muitos estudos sobre a ocorrência de complicações de diabetes, ainda são escassos os de base populacional que abordam sua relação com o tempo de diagnóstico. Ressalta-se a importância de avaliar a ocorrência do diabetes mellitus em relação à utilização de serviços de saúde, sejam estes públicos ou privados, tendo em vista que a maior continuidade do cuidado ^15^, bem como o número de consultas na APS, estão associados ao menor risco de internação pelas condições sensíveis a APS no Sistema Único de Saúde (SUS) ^16^. 

No âmbito do sistema de saúde privado, dados da Agência Nacional de Saúde (ANS) mostraram que em 2021 aproximadamente 46 milhões de brasileiros (25%) eram beneficiários de planos de assistência médica ^17^. A taxa de cobertura, no entanto, apresentou grandes diferenças entre as regiões do país, sendo maior no Sudeste, Sul e Centro-oeste (36,2%; 25,3% e 22,9%, respectivamente) e menor no Nordeste (12,6%) e Norte (11,1%) ^17^. Essa população era composta majoritariamente por pessoas economicamente ativas e com maior taxa de utilização de serviços de saúde, comparativamente aos que não possuíam planos de saúde ^18^. Nos serviços públicos, a Estratégia Saúde da Família (ESF) propiciou o fortalecimento da APS, no entanto, o subfinanciamento do SUS pode comprometer sua capacidade de assegurar a qualidade do cuidado e o acesso integral da população aos serviços de saúde que necessita ^18^ num contexto em que cerca de 70% da população brasileira não possui plano privado de saúde. Ressalta-se que o diabetes mellitus é uma condição em que a APS tem papel fundamental na prevenção, no diagnóstico, no controle e no cuidado ^19^.

Assim, o objetivo deste estudo foi estimar a ocorrência de complicações do diabetes mellitus em pessoas com idade ≥ 40 anos e sua associação com o tempo de diagnóstico da doença, acesso a plano de saúde, uso de serviços de saúde e de medicamentos. 

## Métodos

Este estudo transversal de base populacional utilizou dados de domínio público da PNS 2019, - um inquérito representativo da população com idade ≥ 15 anos, residente em domicílios particulares permanentes no Brasil ^20^ ^,^ ^21^. 

A amostra da PNS foi uma subamostra probabilística da amostra mestra da *Pesquisa Nacional por Amostra de Domicílios* (PNAD) do Instituto Brasileiro de Geografia e Estatística (IBGE), obtida em três estágios. Na primeira etapa, as unidades primárias de amostragem (UPA) foram selecionadas por amostragem aleatória simples. Na segunda, um número fixo de domicílios particulares permanentes foi selecionado por amostragem aleatória simples dentro de cada UPA sorteada. Na terceira etapa, um morador com 18 anos ou mais, que foi sorteado de uma lista de moradores obtida no momento da entrevista, respondeu ao questionário individual. Em 2019, foram visitados 100.541 domicílios e realizadas 94.114 entrevistas domiciliares, além de 90.846 entrevistas individuais. A taxa de não resposta foi de 6,4%, e a coleta de dados ocorreu entre os meses de agosto de 2019 e março de 2020 ^20^ ^,^ ^21^.

Neste estudo, foram considerados dados de indivíduos com idade ≥ 40 anos (n = 54.987). Estimou-se a prevalência de diagnóstico autorreferido de diabetes mellitus pela pergunta: “Algum médico já lhe disse que você tem diabetes?” (sim; não). Entre os indivíduos que responderam sim (n = 6.821), foi estimado o tempo de diagnóstico da doença, gerando-se uma variável quantitativa (medida em anos completos) e obtida pela diferença da idade do indivíduo na data da entrevista e da sua idade na data do diagnóstico. Em seguida, o tempo de diagnóstico da doença foi categorizado de acordo com a distribuição em quartis: de 0-3 anos, de 4-8 anos, de 9-17 anos e 18 anos ou mais de duração.

Entre as pessoas com diabetes, foi verificada a ocorrência de complicações da doença a partir da pergunta: “O(a) Sr(a) tem ou teve alguma destas complicações por causa do diabetes?”; com as seguintes opções de respostas: problema na vista; infarto ou AVC/derrame ou outro problema circulatório; problema nos rins; úlcera/ferida nos pés ou amputação de membros (pés, pernas, mãos ou braços); coma diabético; outro. A prevalência total de complicações foi estimada considerando os indivíduos que responderam positivamente para qualquer uma das opções de resposta. Neste estudo, as opções “coma diabético” e “outro” não foram incluídas, pois o número de observações foi insuficiente para estimativas confiáveis. 

A utilização de plano de saúde foi verificada pela pergunta: “Tem algum plano de saúde médico particular, de empresa ou órgão público?” (sim; não); e o uso de serviços de saúde pela questão: “Quando foi a última vez que o(a) Sr(a) recebeu atendimento médico por causa do diabetes?” (menos de 6 meses; de 6 meses a menos de 1 ano; de 1 ano a menos de 2 anos; de 2 anos a menos de 3 anos; 3 anos ou mais; nunca fez). Devido ao número de observações, agruparam-se as categorias em: (1) < 6 meses; (2) 6 meses < 3 anos; e a categoria “nunca fez” foi excluída devido a nenhuma ocorrência. O agrupamento considerado para a variável “uso de serviços” é compatível com os protocolos do Ministério da Saúde vigentes para o cuidado das pessoas com diabetes, que recomendam atendimento em serviços de saúde com frequência mínima de duas vezes ao ano, para monitoramento das metas glicêmicas estabelecidas ^12^. 

Para as variáveis supracitadas, foram estimadas as prevalências e os respectivos intervalos de 95% de confiança (IC95%) e verificadas as diferenças entre as proporções pelo teste χ^2^ de Pearson (Rao-Scott). As associações entre a ocorrência das complicações selecionadas e o tempo de diagnóstico do diabetes, ter um plano de saúde e a frequência de atendimento médico para o diabetes foram verificadas por meio das razões de prevalência (RP) brutas e ajustadas (por sexo, idade, escolaridade e região de residência) e IC95%, estimadas pela regressão de Poisson com variância robusta. 

Também foi estimada a frequência de utilização de serviço de saúde para acompanhamento do diabetes de acordo com a presença de complicações (com; sem), o tempo de diagnóstico da doença em quartis, plano de saúde (com; sem) e o uso de medicamentos (está usando; não está usando) a partir da pergunta: “O(A) Sr(a) vai ao médico/serviço de saúde regularmente para acompanhamento do diabetes?” (sim; não). As diferenças foram verificadas pelo teste χ^2^ de Pearson (Rao-Scott). 

Além disso, estimou-se as prevalências de uso de medicamento (oral ou insulina) para tratamento do diabetes entre as pessoas sem e com alguma complicação decorrente da doença (independente da complicação). A variável “uso de medicamentos para o tratamento do diabetes” foi gerada considerando como resposta positiva os indivíduos que responderam “sim” para uma ou ambas as perguntas: “Nas duas últimas semanas, por causa do diabetes, o(a) Sr(a) tomou os medicamentos orais para baixar o açúcar?” ou “Usou a insulina receitada na última prescrição?”. Entre as pessoas que responderam “não”, foi verificado o motivo de não ter usado o medicamento oral ou a insulina prescrita pelo médico: “Qual o principal motivo de não ter tomado todos os medicamentos orais receitados para controlar o diabetes?/Qual o principal motivo de não ter usado a insulina para controlar o diabetes?”. 

Adicionalmente, foram estimados os números absolutos das pessoas com as complicações supracitadas - e daquelas que relataram não fazer uso dos medicamentos prescritos -, utilizando o comando *svy* total para dimensionar o número efetivo de indivíduos em tais condições. Todas as análises foram executadas no software Stata (https://www.stata.com), versão 16 , empregando-se o módulo *survey*, para amostras complexas. Em todas as análises, foram considerados níveis de 5% de significância. 

## Resultados 

**Figura 1:**
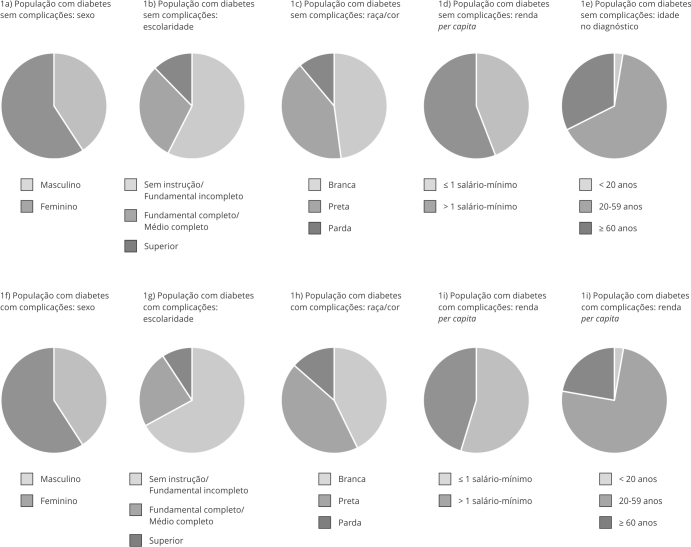
Distribuição da população (idade ≥ 40 anos) com diagnóstico de diabetes sem * e com ** complicações, de acordo com as características sociodemográficas e a idade do diagnóstico. *Pesquisa Nacional de Saúde*, Brasil, 2019.

A prevalência de diabetes mellitus em pessoas com idade ≥ 40 anos foi de 13,4% (IC95%: 12,9-13,9) e a prevalência geral de complicações da doença foi de 31% (IC95%: 29,2-32,9). A Figura 1 ilustra a distribuição da população com diagnóstico de diabetes sem (Figuras 1a, 1b, 1c, 1d e 1e) e com (Figuras 1f 1g, 1h, 1i e 1j) complicações, de acordo com as características sociodemográficas. Embora a maioria dos participantes apresentasse nenhuma instrução ou com Ensino Fundamental incompleto e a proporção de pessoas pretas e pardas tenha sido maior em ambos os estratos, a escolaridade e a proporção de pessoas brancas foram maiores no grupo sem complicações (p < 0,001; p = 0,029). Observou-se diferença na renda per capita: a maioria das pessoas sem complicações apresentou renda superior a um salário mínimo, ao passo que a renda foi menor nas pessoas com complicações da doença (p < 0,001). Quanto à idade no diagnóstico, em ambos os grupos a maioria referiu ter descoberto a doença entre 20 e 59 anos de idade, no entanto a proporção de pessoas que recebeu o diagnóstico com 60 anos ou mais foi maior entre aquelas sem complicações do diabetes (p < 0,001) (Figura 1). 

* População com diabetes sem complicações, n = 4.574; ** População com diabetes com complicações, n = 2.247.

As complicações mais prevalentes foram problemas de visão e nos rins, infarto/AVC/outro problema circulatório, seguido de úlceras/feridas nos pés ou amputação de membros. Observou-se que as prevalências de todas as complicações aumentaram em relação ao tempo de diagnóstico da doença: problemas de visão passaram de 23,5% para 35,8% e a ocorrência de infarto/AVC/outros problemas circulatórios mais que dobrou entre as pessoas com diabetes há 18 anos ou mais - comparativamente àquelas com 0-3 anos de diagnóstico. A frequência de problemas nos rins e úlceras/feridas nos pés ou amputações aumentou cerca de três vezes ao comparar os mesmos grupos (Tabela 1). 

**Tabela 1:** Prevalência de complicações do diabetes na população (idade ≥ 40 anos), segundo tempo de diagnóstico da doença, ter plano de saúde e utilização de serviços de saúde. *Pesquisa Nacional de Saúde*, Brasil, 2019.

Variáveis e categorias	Problema de visão		Infarto/AVC/Outro problema circulatório		Problema nos rins		Úlcera/Ferida nos pés ou amputação de membro	
	%	IC95%	%	IC95%	%	IC95%	%	IC95%
Tempo de diagnóstico do diabetes (anos)		p < 0,001		p = 0,015		p < 0,001		p < 0,001
0-3	23,5	19,6-27,9	5,1	2,4-10,3	5,0	3,6-6,8	3,6	2,3-5,4
4-8	29,3	25,4-33,5	5,5	3,7-8,1	6,7	5,2-8,7	3,3	2,3-4,6
9-17	30,3	27,0-34,0	6,7	5,1-8,7	10,3	8,3-12,7	6,9	5,2-8,9
18 ou mais	35,8	31,7-40,0	11,9	9,0-15,5	15,1	12,4-18,4	9,1	7,1-11,7
Posse de plano de saúde		p < 0,001		p = 0,138		p = 0,360		p = 0,889
Sim	21,5	18,5-25,0	5,8	4,1-8,1	8,4	6,6-10,6	5,6	3,9-8,0
Não	32,7	30,3-35,2	7,9	6,2-10,0	9,6	8,3-11,0	5,7	4,9-6,8
Última vez que recebeu atendimento médico por causa do diabetes		p = 0,007		p = 0,084		p = 0,103		p = 0,106
< 6 meses	31,1	28,8-33,6	7,9	6,2-10,0	9,8	8,5-11,3	6,1	5,1-7,3
6 meses < 3 anos	25,2	21,9-28,8	5,6	4,0-7,7	7,7	5,9-9,9	4,6	3,4-6,2
Total	29,6	27,6-31,6	7,3	6,0-8,9	9,2	8,2-10,4	5,7	4,9-6,6

AVC: acidente vascular cerebral; IC95%: intervalo de 95% de confiança.

Quanto à posse de plano de saúde, foi verificada diferença significativa em relação aos problemas de visão, cuja ocorrência foi maior nos indivíduos sem plano (32,7% *vs.* 21,5%; p < 0,001). No que se refere à frequência de atendimento médico por causa do diabetes, maior prevalência de problema de visão foi observada entre as pessoas que tinham ido ao médico nos últimos seis meses em relação às que foram de 6 meses < 3 anos (31,1% *vs.* 25,2%; p = 0,007), sem diferença significativa para as demais complicações (Tabela 1).

Foi observada a associação entre o tempo de diagnóstico e a ocorrência de complicações: na comparação das pessoas com 18 anos ou mais, em relação àquelas com até 3 anos de diagnóstico, observaram-se maiores ocorrências de problemas de visão (RP ajustada = 1,8; IC95%: 1,4-2,2), infarto/AVC/outro problema circulatório (RP ajustada = 2,2; IC95%: 1,1-4,3), problemas nos rins (RP ajustada = 3,4; IC95%: 2,3-5,2) e de úlceras/feridas nos pés ou amputação de membros (RP ajustada = 2,6; IC95%: 1,5-4,5) (Tabela 2). 

**Tabela 2:** Razões de prevalência brutas e ajustadas por sexo, idade, escolaridade e região de residência para a ocorrência de complicações do diabetes na população com idade ≥ 40 anos, segundo: tempo de diagnóstico da doença, ter plano de saúde e utilização de serviços de saúde. *Pesquisa Nacional de Saúde*, Brasil, 2019.

Variáveis e categorias	Problemas na vista		Infarto/AVC/ outro problema circulatório		Problema nos rins		Úlcera/feridas nos pés ou amputação de membros	
	RP bruta	RP ajustada (IC95%)	RP bruta	RP ajustada (IC95%)	RP bruta	RP ajustada (IC95%)	RP bruta	RP ajustada (IC95%)
Tempo de diagnóstico do diabetes (anos)								
0-3	1,0	1,0	1,0	1,0	1,0	1,0	1,0	1,0
4-8	1,2 (1,0-1,6)	1,3 (1,1-1,6)	1,1 (0,5-2,5)	1,1 (0,5-2,4)	1,4 (0,9-2,0)	1,4 (0,9-2,1)	0,9 (0,5-1,6)	0,9 (0,5-1,6)
9-17	1,3 (1,0-1,6)	1,4 (1,1-1,7)	1,3 (0,6-2,9)	1,3 (0,6-2,6)	2,1 1,4-3,1	2,2 (1,5-3,4)	1,9 (1,1-3,2)	1,9 (1,1-3,3)
18 ou mais	1,5 (1,2-1,9)	1,8 (1,4-2,2)	2,3 (1,1-5,1)	2,2 (1,1-4,3)	3,1 (2,1-4,5)	3,4 (2,3-5,2)	2,5 (1,5-4,2)	2,6 (1,5-4,5)
Posse de plano de saúde								
Não	1,0	1,0	1,0	1,0	1,0	1,0	1,0	1,0
Sim	0,6 (0,5-0,8)	0,7 (0,6-0,9)	0,7 (0,5-1,1)	0,7 (0,5-1,0)	0,9 (0,7-1,1)	1,0 (0,7-1,4)	1,0 (0,6-1,4)	0,9 (0,6-1,4)
Última vez que recebeu atendimento médico por causa do diabetes								
< 6 meses	1,0	1,0	1,0	1,0	1,0	1,0	1,0	1,0
6 meses < 3 anos	0,8 (0,7-0,9)	0,8 (0,7-0,9)	0,7 (0,5-1,0)	0,7 (0,5-1,0)	0,8 (0,6-1,0)	0,8 (0,6-1,0)	0,7 (0,5-1,1)	0,7 (0,5-1,1)

AVC: acidente vascular cerebral; IC95%: intervalo de 95% de confiança; RP: razão de prevalência.

**Figura 2:**
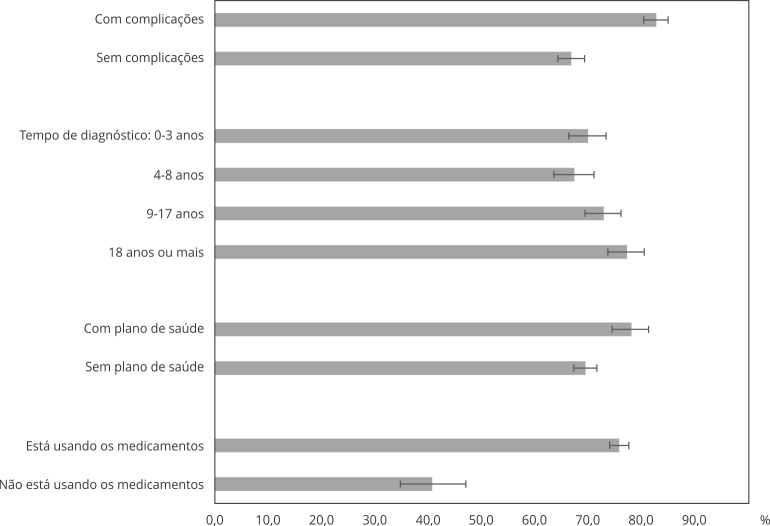
Prevalência de uso de serviços pela população com idade ≥ 40 anos para acompanhamento do diabetes, de acordo com a presença de complicação, o tempo de diagnóstico da doença (em anos), ter de plano de saúde e o uso de medicamentos para tratamento da doença. *Pesquisa Nacional de Saúde*, Brasil, 2019.

O uso de serviços de saúde para o acompanhamento do diabetes mellitus foi maior nas pessoas com complicações (82,9%; IC95%: 80,5-85,1) em relação àquelas sem complicações (66,9%; IC95%: 64,4-69,4; p < 0,001) e aumentou em função do tempo de diagnóstico da doença, de 70,1% (IC95%: 66,4-73,5) nos indivíduos com até 3 anos de diagnóstico para 77,4% (IC95%: 73,8-80,6) naqueles com 18 anos ou mais de diagnóstico (p < 0,001) (Figura 2).

O uso dos serviços também foi mais referido pelas pessoas com plano de saúde (78,2%; IC95%: 74,6-81,4) comparativamente às pessoas sem plano (69,6%; IC95%: 67,4-71,7), bem como entre as pessoas que estavam tomando os medicamentos prescritos (75,9%; IC95%: 74,1-77,7), em relação as que referiram não aderir ao tratamento medicamentoso (40,8%; IC95%: 34,8-47,1) (Figura 2).

A prevalência do uso de medicamentos (oral ou insulina) nas pessoas sem complicações do diabetes foi de 84,1% (IC95%: 82,1-85,8), e nas pessoas com complicações atingiu 93,3% (IC95%: 91,5-94,8). Entre as pessoas com indicação de tratamento farmacológico, mas que não faziam uso dos medicamentos prescritos, os principais motivos para a não adesão foram: “não precisar mais tomar o medicamento porque o diabetes estava controlado”, referido por 41,4% (IC95%: 35,1-48,1) dos usuários com prescrição de medicamentos orais e por 67,5% (IC95%: 63,5-71,3) dos usuários de insulina; seguido pelo motivo “não achar necessário”, reportado por 37,4% (IC95%: 32,3-42,8) das pessoas com prescrição de medicamentos orais e por 21,6% (IC95%: 18,3-25,2) dos usuários de insulina (dados não apresentados em tabela). 

**Figura 3:**
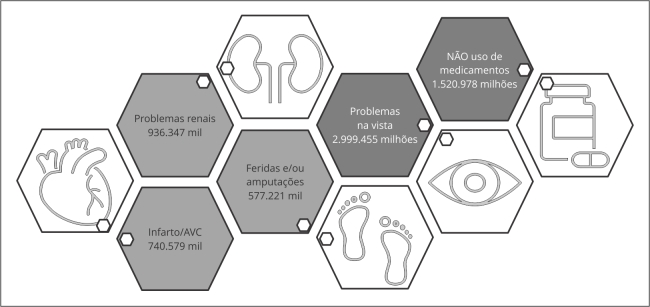
Estimativa do número absoluto de pessoas (idade ≥ 40 anos) com diagnóstico de diabetes e complicações decorrentes da doença, e número de pessoas com indicação de tratamento medicamentoso (oral ou insulina) que não faziam uso do medicamento prescrito. *Pesquisa Nacional de Saúde*, Brasil, 2019.

A Figura 3 apresenta as estimativas do número absoluto total de adultos brasileiros (idade ≥ 40 anos) com diabetes e com indicação de tratamento farmacológico, mas que não faziam uso de medicamentos - mais de 1,5 milhão de pessoas. Além disso, as estimativas apontaram que, em 2019, quase três milhões de pessoas com diabetes apresentaram problemas de visão e mais de 936 mil problemas renais. A ocorrência de infarto/AVC/outros problemas circulatórios atingiu 740.579 mil pessoas e 577.221 mil sofreram com feridas e/ou amputações. 

AVC: acidente vascular cerebral.

## Discussão

Os resultados do estudo mostraram que as complicações decorrentes do diabetes mais prevalentes foram problemas de visão e nos rins, seguidas de infarto/AVC/outro problema circulatório e úlceras/feridas nos pés ou amputação de membros - e que três em cada dez pessoas com diabetes tem ao menos uma complicação. O tempo de diagnóstico das doenças esteve associado ao aumento das prevalências de todas as complicações do diabetes. Particularmente, os problemas de visão foram mais prevalentes entre os indivíduos sem plano de saúde e nos que procuravam atendimento médico mais recentemente (até 6 meses). Nas pessoas com diabetes e idade ≥ 40 anos, a utilização de serviços de saúde aumentou em função da presença de complicações, do tempo de diagnóstico da doença, de ter plano de saúde e do uso de medicamentos. Quanto ao uso de medicamentos, embora tenham sido observadas elevadas proporções entre aqueles com diabetes e ao menos uma complicação da doença, mais de 1,5 milhão de pessoas com indicação de uso não o fazem. 

No que se refere às complicações da doença, estudo recente, também com dados da PNS 2019, destacou que aproximadamente uma em cada dez pessoas com diabetes apresentaram duas ou mais complicações decorrentes da doença. Esse estudo abordou as complicações do diabetes mellitus sob a perspectiva das desigualdades sociais, avaliando escolaridade e renda, e identificou aumento da ocorrência de complicações entre pessoas com menores níveis socioeconômicos ^4^. Outro estudo com dados da PNS 2013 mostrou que as complicações mais prevalentes foram: problemas de visão, seguidos de problema circulatório periférico e nos rins ^8^. Esses resultados são semelhantes aos encontrados neste estudo, no qual as complicações mais prevalente foram problemas na vista, seguido de problemas nos rins e circulatórios.

De modo particular, os problemas de visão decorrentes do diabetes (retinopatia diabética, glaucoma, catarata, que podem evoluir para perda total da visão) ^5^ ^,^ ^12^ têm impacto significativo na qualidade de vida das pessoas acometidas. Por serem assintomáticos nos estágios iniciais, é essencial que as pessoas com diabetes façam exames regulares de rastreamento para detecção desses problemas, como retinopatia diabética e edema macular diabético ^5^. 

Este estudo encontrou maior proporção de problemas de visão em pessoas sem plano de saúde em relação aos usuários com plano de saúde. Este resultado pode estar relacionado à baixa cobertura de exames dos olhos no SUS, conforme observado em estudos anteriores ^8^ ^,^ ^22^ ^,^ ^23^. Além disso, os usuários de planos privados podem acessar diretamente oftalmologistas, ao passo que, na APS do SUS, é recomendada a realização de teste de acuidade visual (teste de Snellen) em pessoas com diabetes mellitus para estratificação e encaminhamento ao oftalmologista uma vez ao ano, ou antes, a depender da necessidade, considerando a classificação de risco do teste de Snellen ^12^. 

Neste estudo, a ocorrência de complicações foi maior nas pessoas com diagnóstico de diabetes há mais tempo. Não foram encontrados estudos nacionais de base populacional que investigaram essa associação. Estudo realizado com dados do sistema público de cadastramento de pessoas com hipertensão e diabetes de um município de médio porte no centro-oeste de Minas Gerais verificou que, entre os usuários que possuíam o diagnóstico de diabetes há mais de 10 anos, o percentual dos que apresentaram complicações (32,2%) foi maior do que os que tinham diagnóstico da doença entre 5 e 10 anos (14,2%) e menos que cinco anos (12,1%) ^14^, demonstrando que a ocorrência de complicações aumentou em relação ao tempo de diagnóstico. Esses resultados corroboram com os encontrados neste estudo - que mostram maiores proporções de complicações à medida que o tempo de diagnóstico aumenta -, embora o recorte temporal utilizado seja distinto: a prevalência geral de complicações foi de 30,1% nas pessoas com diagnóstico há 18 anos ou mais, de 24,6% entre 9 e 17 anos e 22,4% nas pessoas com diagnóstico há menos de nove anos (dados não apresentados). Cabe ressaltar que as complicações do diabetes podem evoluir gradualmente ao longo da vida, resultando, principalmente, do estreitamento dos vasos sanguíneos devido a maior exposição a níveis elevados de glicemia ^24^. 

Em consonância com os resultados encontrados neste estudo, no cenário internacional, um estudo realizado na Irlanda verificou aumento da prevalência de diabetes mellitus entre pessoas com 40 anos e mais, principalmente naquelas com idade ≥ 70 anos, com reflexos no aumento de complicações ^25^. Outra pesquisa realizada em uma clínica para tratamento do diabetes em Verona, na Itália, identificou a ocorrência de complicações crônicas da doença, micro e macro vasculares ^26^. Ainda, um estudo mexicano com dados da PNS 2013 no Brasil e da *Pesquisa Nacional de Saúde e Nutrição* de 2018 no México avaliou a chance de desenvolver complicações em relação ao tempo vivendo com diabetes - no conjunto da população de ambos os países, não permitindo a separação entre brasileiros e mexicanos nesta análise. Verificou-se que participantes com histórico de diabetes há mais tempo apresentaram maiores chances de ter complicações ^27^. 

O maior número de consultas na APS está associado a menores proporções de internações por condições sensíveis, como o diabetes ^16^ ^,^ ^28^. Um estudo que avaliou indicadores da linha de cuidado de diabetes mellitus no Brasil, com dados da PNS de 2019, mostrou que as internações por causa do diabetes e de suas complicações são mais frequentes nas pessoas com menores proporções de uso dos serviços de saúde e de realização de exames de rastreamento ^28^. No presente estudo, maiores prevalências de problemas de visão foram observadas entre as pessoas que visitaram o médico nos últimos seis meses em relação aos que ultrapassaram esse tempo (31,1% *vs.* 25,2%). Embora os estudos transversais não permitam avaliar relações temporais ^29^, outros resultados deste estudo - como a maior prevalência de uso dos serviços de saúde entre as pessoas com complicações e naquelas que aderiram o tratamento farmacológico prescrito - podem indicar que as pessoas que procuram os serviços de saúde com maior frequência tenham maior adesão aos cuidados propostos justamente por serem acometidas por este problema. 

Ressalta-se que a *Portaria nº 483/2014* do Ministério da Saúde ^30^ estabeleceu as diretrizes para organização das linhas de cuidado à saúde das pessoas com doenças crônicas no âmbito do SUS e definiu como estratégia a realização de exames de rastreamento, ações de prevenção, diagnóstico e tratamento precoce de possíveis complicações da doença. Para isso, além de garantir o acesso à consulta, é necessário que o cuidado seja integral, com diagnóstico em tempo oportuno, realização de exames de rastreamento - de modo a detectar precocemente complicações decorrentes da doença - e promoção de intervenções em tempo hábil para diminuir a sobrecarga dos serviços especializados ^8^ em função do agravamento das complicações.

Este estudo encontrou elevada frequência de uso de medicamentos nas pessoas com diabetes mellitus sem complicações (84,1%) e entre aquelas com complicações decorrentes da doença (93,3%). Ainda assim, é preciso considerar o grande número de pessoas que não fazem uso mesmo com a indicação, correspondendo a mais de 1,5 milhão de brasileiros de acordo com estimativas obtidas neste estudo. Pessoas com diabetes podem ter dificuldade em aderir ao tratamento devido a uma variedade de causas, como fatores pessoais, problemas relacionados à organização e preparação de medicamentos, problemas financeiros e falhas ou deficiências nas práticas dos profissionais e equipes de saúde ^31^. 

Considerando os motivos referidos pelas pessoas com a doença que não usavam medicamento à época da pesquisa - não precisar mais tomar o medicamento porque o diabetes estava controlado e não achar necessário -, é possível identificar o baixo letramento funcional em saúde (a capacidade cognitiva de entender, interpretar e aplicar informações escritas ou faladas sobre saúde) e tal fato está associado à baixa adesão ao tratamento medicamentoso e tem relação com a escolaridade, renda e cor da pele ^32^ ^,^ ^33^. Portanto, o baixo letramento funcional e a consequente baixa adesão ao tratamento prescrito contribuem para o aumento das complicações da doença. Essa relação mostra o impacto das desigualdades sociais na capacidade de autocuidado das pessoas com diabetes. Além disso, devido ao estigma do diabetes na mídia e na sociedade, a condição é, por vezes, atribuída simplesmente a dietas e estilos de vida não saudáveis, ignorando fatores genéticos, regionais, econômicos e sociais. Essa visão simplista contribui para que os pacientes não recebam o cuidado necessário e enfrentem prejuízos psicológicos e sociais significativos ^3^.

Nesse contexto, destaca-se a importância da preparação dos profissionais de saúde para atender a essa população e orientarem os usuários, considerando seu conhecimento acerca do diabetes mellitus e as suas necessidades específicas, visando à melhoria da adesão ao tratamento e assim obtendo melhores resultados adequados à saúde. Devem ser desenvolvidas estratégias de forma integrada e contínua, a fim de ajudar a aumentar a compreensão do paciente sobre o diabetes e sua potencialidade no controle da doença, melhorando o comprometimento com o tratamento e a prevenção de complicações, tendo em vista que a maioria dos cuidados com a saúde depende dos níveis de autogerenciamento dos usuários ^34^.

Devido ao aumento na prevalência da doença, os serviços do SUS têm se mobilizado com foco na atenção preventiva e promoção à saúde desta população em risco, investindo em serviços de atendimento específicos, fornecimento de medicamentos e insulina de forma gratuita, exames e capacitação profissional para lidar com o diabetes mellitus, dentre outras doenças crônicas ^12^ ^,^ ^35^ ^,^ ^36^.

Entre as limitações deste estudo, deve-se considerar que todas as informações foram referidas, permitindo subestimação do tempo de diagnóstico da doença, além de viés de memória quanto ao último atendimento. Ainda, as complicações autorreferidas, como por exemplo “problemas nas vistas”, podem ter um viés de aferição maior que as relacionadas ao pé diabético. Este estudo apresenta como pontos fortes o uso de dados de base populacional, com foco no tempo de diagnóstico - além de analisar o uso de serviços de saúde e uso de medicamentos: em geral, estudos de base populacional sobre complicações do diabetes no Brasil são escassos, particularmente considerando o aspecto adotado neste estudo. Cabe ressaltar que não foi encontrado nas bases de dados nenhum estudo nacional de base populacional que avaliou a associação entre tempo de diagnóstico e ocorrência de complicações na população brasileira. 

Com o aumento de casos de diabetes mellitus e da ocorrência de diferentes complicações associadas, faz-se necessário o fortalecimento das recomendações e ações do SUS, em especial da APS para identificação, controle e tratamento do diabetes mellitus e exames de rastreamento de complicações - para os quais existem embasamentos, mas ainda não são devidamente implementados na prática em saúde ^8^ ^,^ ^12^. A qualidade e a expectativa de vida da população brasileira está intimamente ligada à existência, monitoramento e controle das DCNT que vêm se sobressaindo com a transição demográfica evidenciada em recentes pesquisas ^37^ ^,^ ^38^. 

Em conclusão, os achados mostraram que, dentre todas as complicações, os problemas de visão e nos rins foram as principais provocadas pelo diabetes mellitus - assim como a utilização de serviços de saúde estiveram diretamente relacionadas com o tempo de diagnóstico da doença. Os problemas de visão foram mais frequentes nos indivíduos sem plano médico de saúde e nos que buscaram atendimento médico nos últimos seis meses e que, embora o uso de medicamentos tenha sido mais elevado nos adultos com diabetes mellitus e com presença de alguma complicação da doença, mais de 1,5 milhão de pessoas neste grupo não faziam uso dos medicamentos prescritos, gerando importante impacto para a qualidade de vida e para os serviços de saúde, família e sociedade.
